# Application of Meta-Analysis and Machine Learning Methods to the Prediction of Methane Production from In Vitro Mixed Ruminal Micro-Organism Fermentation

**DOI:** 10.3390/ani10040720

**Published:** 2020-04-21

**Authors:** Jennifer L. Ellis, Héctor Alaiz-Moretón, Alberto Navarro-Villa, Emma J. McGeough, Peter Purcell, Christopher D. Powell, Padraig O’Kiely, James France, Secundino López

**Affiliations:** 1 Centre for Nutrition Modelling, Department of Animal Biosciences, University of Guelph, 50 Stone Road East, Guelph, ON N1G 2W1, Canada; cpowell@uoguelph.ca (C.D.P.); jfrance@uoguelph.ca (J.F.); 2 Departamento de Ingeniería Eléctrica de Sistemas y Automática, Escuela de Ingeniería Industrial e Informática, Universidad de León, Campus Universitario de Vegazana, 24071 León, Spain; hector.moreton@unileon.es; 3 Animal & Grassland Research and Innovation Centre, Teagasc, Grange, Dunsany, Co. Meath C15 PW93, Ireland; alberto.navarro.villa@trouwnutrition.com (A.N.-V.); emma.mcgeough@umanitoba.ca (E.J.M.); peter.purcell86@gmail.com (P.P.); Padraig.OKiely@teagasc.ie (P.O.); 4 Trouw Nutrition R&D, Ctra. CM-4004 km 10.5, 45950 El Viso de San Juan, Spain; 5 Department of Animal Science, University of Manitoba, Winnipeg, MB R3T 2N2, Canada; 6 Instituto de Ganadería de Montaña (IGM), CSIC-Universidad de León, Departamento de Producción Animal, Universidad de León, 24007 León, Spain

**Keywords:** in vitro gas production, methane, rumen, feed, meta-analysis, machine learning, neural network

## Abstract

**Simple Summary:**

In vitro gas production systems are regularly utilized to screen feed ingredients for inclusion in ruminant diets. However, not all in vitro systems are set up to measure methane (CH_4_) production, nor do all papers report in vitro CH_4_. Therefore, the objective of this study was to develop models to predict in vitro production of CH_4_, a greenhouse gas produced by ruminants, from in vitro gas and volatile fatty acid (VFA) production data, and to identify the major drivers of CH_4_ production in these systems. Meta-analysis and machine learning (ML) methodologies were applied to predict CH_4_ production from in vitro gas parameters. Meta-analysis results indicate that equations containing apparent dry matter (DM) digestibility, total VFA production, propionate, valerate and feed type (forage vs. concentrate) resulted in best prediction of CH_4_. The ML models far exceeded the predictability achieved using meta-analysis, but further evaluation on an external database would be required to assess their generalization capacity. The models developed can be utilized to estimate CH_4_ emissions in vitro.

**Abstract:**

In vitro gas production systems are utilized to screen feed ingredients for inclusion in ruminant diets. However, not all in vitro systems are set up to measure methane (CH_4_) production, nor do all publications report in vitro CH_4_. Therefore, the objective of this study was to develop models to predict in vitro CH_4_ production from total gas and volatile fatty acid (VFA) production data and to identify the major drivers of CH_4_ production in these systems. Meta-analysis and machine learning (ML) methodologies were applied to a database of 354 data points from 11 studies to predict CH_4_ production from total gas production, apparent DM digestibility (DMD), final pH, feed type (forage or concentrate), and acetate, propionate, butyrate and valerate production. Model evaluation was performed on an internal dataset of 107 data points. Meta-analysis results indicate that equations containing DMD, total VFA production, propionate, feed type and valerate resulted in best predictability of CH_4_ on the internal evaluation dataset. The ML models far exceeded the predictability achieved using meta-analysis, but further evaluation on an external database would be required to assess generalization ability on unrelated data. Between the ML methodologies assessed, artificial neural networks and support vector regression resulted in very similar predictability, but differed in fitting, as assessed by behaviour analysis. The models developed can be utilized to estimate CH_4_ emissions in vitro.

## 1. Introduction

Globally, greenhouse gas (GHG) emissions from the agriculture, forestry and other land use (AFOL) sector account for ~23% of the global anthropogenic GHG total emissions [1], with enteric methane (CH_4_) from fermentation in the forestomach of ruminants representing 32%–40% of that total [1] (thereby 7.4%–9.2% of the global anthropogenic total). From the farmer’s perspective, CH_4_ also represents an energy loss and an inefficiency of production, ranging from approximately 3.0 (feedlot cattle) to 7.0 (forage fed cattle) percent of gross energy intake, with a ±20% uncertainty [2]. As a result, and to meet public expectation for sustainably produced food products, the agriculture sector has mobilized to examine a large array of potential CH_4_ (as well as N and P excretion) mitigation strategies [3,4,5], to reduce the environmental impact of livestock and food production.

At the animal level, CH_4_ is produced as a byproduct of anaerobic fermentation in the rumen and hindgut of ruminants, whereby methanogens utilize H_2_ to obtain ATP by reducing CO_2_ to CH_4_ [6]. The removal of H_2_ through methanogenesis, the main H-sink in the rumen [6], prevents the inhibitory effect of H_2_ on ruminal fermentation and allows for the degradation and fermentation of feed to proceed. When methanogenesis is reduced, other pathways must be promoted to utilize H_2_ or otherwise fermentation, digestibility and intake may be negatively affected [6].

As animal experiments to evaluate feedstuffs and feed additives are costly, time consuming and do not guarantee conclusive outcomes, the in vitro gas production technique represents a viable option for prescreening or screening of feedstuffs/additives for potential inclusion in the ration of modern dairy cows, beef cattle and other ruminants. However, CH_4_ is often, but not always, included in the gases measured during in vitro incubation (particularly in developing countries where equipment may be unaffordable, unavailable or limited, for example). A reliable measure of CH_4_ from in vitro cultures of mixed ruminal micro-organisms would be a useful tool to assess the potential dietary effects on methanogenesis. Estimation of CH_4_ from the output of other fermentation end-products commonly measured in vitro could be a suitable alternative, and Jayanegara [7] proposed the use of the stoichiometric equations of Hegarty and Nolan [8] and of Moss et al. [9] to predict in vitro CH_4_. However, using this approach CH_4_ was generally overpredicted, presumably because in vitro H_2_ recovery observed in practice was substantially less than that assumed by the stoichiometric models. The objectives of this study were therefore to: (1) to develop empirical models to predict in vitro CH_4_ production from in vitro gas production measures—via meta-analysis (multiple linear regression) and machine learning (ML) methods (artificial neural networks, ANN, and support vector regression, SVR), and (2) to identify the fermentation parameters most closely related to CH_4_ production in vitro. 

## 2. Materials and Methods

### 2.1. Database

The database compiled for this study consisted of 397 in vitro rumen fermentation bottle means (each the average of 3–5 replicate measurements), taken after 24 h of incubation, from 13 experiments reported in 10 publications [10,11,12,13,14,15,16,17,18,19] (experiments 1–3 were from publication [10]), plus 1 unpublished study [20]. As a result, experimental animals were not directly employed in this study. In accordance with the National Centre for the Replacement Refinement and Reduction of Animals in Research (NC3Rs), per Directive 2010/63/EU, all study data used were publicly available (with the exception of the one unpublished study) as reported in the aforementioned articles. Studies evaluated the in vitro gas and CH_4_ production from oven-dried feedstuffs, including ryegrass, forbs, grass silages, clover, maize silage and other whole-crop cereal silages and concentrate feeds (no feed additives or rumen modifiers were included in the database). Feed type (FT) was categorized as either forage (FT = 1) or concentrate (FT = 2). The database included in vitro measurements of CH_4_ gas production (CH_4_i, mL/g DM incubated, and CH_4_d, mL/g DM apparently digested), total gas production (TGP, mL/g DM incubated), apparent DM digestibility (DMD, g/g), volatile fatty acid production (VFA, mmol/g DM incubated), molar proportions of acetic acid (AC, mmol/mol VFA), propionic acid (PR, mmol/mol VFA), butyric acid (BT, mmol/mol VFA) and valeric acid (VL, mmol/mol VFA), the acetate to propionate ratio (C2C3) and the incubation medium final pH (pH). Daily production of each volatile fatty acid (ACp, PRp, BTp or VLp for mmol AC, PR, BT or VL produced per g DM incubated, respectively) was calculated from total VFA and the corresponding molar proportions. Variable abbreviations, units and descriptions are also summarized in Appendix A (Table A1). When digestibility is measured during in vitro batch cultures of mixed ruminal micro-organisms, it is assumed that DM disappearance after the incubation time (in this particular case 24 h) is an acceptable metric of apparent DM digestibility. Due to missing data, two studies [11,12], were removed from the database, leaving 354 observations from 11 experiments.

For model development and evaluation purposes, the dataset (*n* = 354) was divided into two subsets, the first one for training and model development purposes (70% of data, *n* = 247, with 4 outlier data points removed for meta-analysis), and the second one for model testing and evaluation (internal evaluation) purposes (30% of data, *n* = 107). Aside from 4 data points which were removed for the meta-analysis (statistical outliers), the ‘two’ developmental datasets were identical. Division of data points into the training or evaluation datasets was via random assignment, but each contained a proportional number of observations relative to the FT variable. Descriptive statistics for the training and evaluation datasets are provided in Table 1.

Independent testing of the model gives a measure of the model’s ‘generalization ability’ (‘test error’), or the ability to make predictions on unseen data. This is particularly important for some ML approaches, which may achieve very accurate predictions, but essentially model the noise in the data. 

### 2.2. Model Fitting—Meta-Analysis

The main effects of in vitro fermentation variables (TGP, DMD, VFA, AC, ACp, PR, PRp, BT, BTp, VL, VLp, C2C3 and final pH) were analyzed for inclusion in predictive models using the PROC MIXED procedure of SAS [21] to predict CH_4_i (mL/g incubated DM), or CH_4_d (mL/g DM apparently digested). Equations were fitted to the training dataset (Table 1—meta-analysis).

The mixed model analysis was chosen because the data were compiled from multiple studies, and thus the experiment was considered as a random effect [22]. If, when running the model, the random covariance or the random slope was not significant, they were removed from the model or simplified [22], though the random intercept term was always retained. The dual quasi-Newton technique was used for optimization with an adaptive Gaussian quadrature as the integration method. Normal distribution of the random study effect was assessed via Q–Q distribution plot, and normality of residuals via examination of the residual plots (PROC MIXED). 

Three approaches were taken to fitting mixed models to this dataset: (1) univariate analysis of each dependent–independent variable combination (explanatory variable in linear, quadratic or cubic form); (2) multivariate analysis, preceded by examination in PROC REG (MaxR) and assessment for collinearity between driving variables in PROC CORR/visual plotting; and (3) multivariate analysis based on known biological principles. Approaches (2) and (3) are not distinguished/presented separately in the results, as both are considered ‘multivariate’. A fourth approach was also included for comparison with the ML models (described below): (4) where all driving variables were included, irrespective of significance or collinearity (linear equation form). With the exception of approach (4), only equations with significant slope parameters (*p* < 0.05) and normally distributed residuals/random effects were retained and evaluated.

### 2.3. Model Fitting—Machine Learning

The in vitro fermentation variables (TGP, DMD, VFA, AC, PR, BT, VL, C2C3 and final pH) were retained as potential driving variables for development of ML-based predictive models for CH_4_i and CH_4_d. Predictive models were fitted on the training dataset (Table 1—Machine learning). The raw dataset was subjected to a preprocessing normalization process (standard scalar) [23] according to:

(1) Z=(X−u)/S

where *Z* is the normalized value, *X* is the raw value, *u* is the mean of the training samples and *S* is the standard deviation of the training samples. The objective of this normalization step was to improve the convergence of the training process in the regression methods utilized [24]. Subsequently, two ML techniques (support vector regression and artificial neural network) were implemented using the Scikit-learn software library [25] for the Python programming language [26]. For both ML approaches, a 10-fold cross-validation procedure was used to fit the predictive models to the training dataset (*n* = 247; the evaluation database, *n* = 107, was therefore not included in this analysis). The training dataset was subsequently randomly split into 10 equal subgroups, and the model was trained using nine of the subsets and validated on the remaining one part of the data to compute a performance measure. This holdout process was repeated for each of the 10-folds, such that each subset was utilized for validation, whereas the other nine subsets were pooled for the training, in turn. The error estimation was averaged over the 10 iterations to assess the fit performance.

#### 2.3.1. Support Vector Regression

Support Vector Machine (SVM) is a ML technique based on supervised learning with a modality oriented for regression problems, namely Support Vector Regression (SVR), able to forecast continuous variables [27] (in this case, CH_4_ from in vitro cultures of mixed ruminal micro-organisms). The SVR method transforms the input data (previously normalized) into a multidimensional space by using nonlinear mapping, and a linear regression procedure is applied to each hyperplane obtained to calculate the desired output. The SVR method is developed by changing the kernel function and tuning the parameters *C* (the regularization parameter), *γ* (the kernel coefficient), Tol (tolerance for stopping criterion) and degree of the polynomial. Three ‘kernel’ functions were considered—linear, radial basis function and polynomial. The ranges of values used for the parameter optimization were *C* ∈ {1, 10, 100, 1000}, Tol ∈ {0.1, 0.01}, *γ* ∈ {1, 0.1, 0.01, 0.001} and degree (only for the polynomial function) ∈ {2, 3, 4, 5, 6}. Grid search combined with cross-validation [27] were used to achieve the best combination of parameters resulting in the optimal and most robust SVR model solution, on the basis of the *ε*-insensitive loss function. The best SVR models for both variables to be predicted (CH_4_i or CH_4_d) were obtained using the radial basis function as kernel, with the parameter values *C* = 1000, Tol = 0.1 and *γ* = 1. 

#### 2.3.2. Artificial Neural Network—Multilayer Perceptron

A multilayer perceptron (MLP) is a machine learning method based on supervised learning, and is a specific topology of a feedforward artificial neural network (ANN) [28]. The MLP network used for the current study was composed of three layers of nodes: the input layer, one hidden layer and an output layer. Achieving the optimal MLP architecture can require tuning a number of hyperparameters such as the number of hidden layers, neurons or iterations. For the current study, one hidden layer was applied and the rectified linear unit (ReLU) nonlinear activation function was implemented in each node (neuron) of this hidden layer (except the input nodes). On the other hand, the single neuron of the output layer utilized the linear activation function [28]. The training procedure was based on the backpropagation technique, using grid search combined with cross-validation [28] to derive the best combination of parameters resulting in the optimal and most robust MLP model solution. The square-error loss function and the limited-memory Broyden–Fletcher–Goldfarb–Shanno (L-BFGS) numerical method were used for optimization. The number of hidden neurons in the best MLP models was 26 and 27 for CH_4_i and CH_4_d predictions, respectively, (the range tested was from 12–30 neurons in the hidden layer). Other hyperparameters were included in the grid for tuning aiming to optimize the training of the ANN. The best results were observed when early stopping was activated (to prevent overfitting), any prior attributes stored on the estimator were cleared (“warm start” disabled), initial learning rate was set at 10^−7^ and kept constant, and the batch size for each iteration was equal to 1.

### 2.4. Model Evaluation

Model predictions developed in the current study (via meta-analysis, SVR or ANN) were evaluated using an independent data subset (internal evaluation, as the data are independent but related to the training dataset), described in Section 2.1 and in Table 1. Models were evaluated for their predictability using mean square prediction error (MSPE), calculated as:

(2) $$\mathrm{MSPE}=\sum_{i=1}^{n}\frac{{\left(O_{i}-P_{i}\right)}^{2}}{n}$$

where *O_i_* is the observed value, *P_i_* is the predicted value and *n* is the number of observations. Square root of the MSPE (RMSPE), expressed as a proportion of the observed mean (RMSPE, %), gives an estimate of the overall prediction error. The RMSPE was decomposed into random (disturbance) error (ED), error due to deviation of the regression slope from unity (ER), and error in central tendency due to overall bias (EB) [29]. The EB, ER and ED fractions of MSPE were calculated as:

(3) $$\mathrm{EB}={\left(\bar{P}-\bar{O}\right)}^{2}$$

(4) $$\mathrm{ER}={\left(S_{p}-R\times S_{o}\right)}^{2}$$

(5) $$\mathrm{ED}=\left(1-R^{2}\right)\times S_{o}{}^{2}$$

where $\bar{P}$ and $\bar{O}$ are the predicted and observed means, *S_p_* is the standard deviation of predicted values, *S_o_* is the standard deviation of observed values and *R* is the Pearson correlation coefficient. 

Correspondence between predicted and observed values was also assessed by the concordance correlation coefficient (CCC) [30], which was calculated as:

(6) $$\mathrm{CCC}=R\times C_{b}$$

where *C_b_* is a bias correction factor (a measure of accuracy), and *R* is the Pearson correlation coefficient (a measure of precision). The *C_b_* variable is calculated as:

(7) $$C_{b}=\frac{2}{\left(\nu +\frac{1}{\nu }+\mu^{2}\right)}$$

where

(8) $$\nu =\frac{S_{o}}{S_{p}}$$

(9) $$\mu =\frac{\bar{O}-\bar{P}}{\sqrt{\left(S_{o}-S_{p}\right)}}$$

so that *ν* provides a measure of scale shift, while *µ* provides a measure of location shift. The *ν* value indicates the change in standard deviation, if any, between predicted and observed values. A positive *µ* value indicates underprediction, while a negative *µ* indicates overprediction. Predictions were further evaluated visually against observations (via predicted vs. observed plots) as well as against residuals (residual vs. predicted, not shown).

As one criticism of many ML methodologies remains their lack of transparency (i.e., no predictive equation is produced), models developed via ANN and SVR were further evaluated using behaviour analysis, where model inputs were systematically altered ±10% (in isolation) and the model’s ‘behavioural’ response (% change in output prediction) was assessed (direction and magnitude).

## 3. Results

### 3.1. Correlation Matrix Analysis

Potential *X* variables were evaluated against each other via correlation matrix analysis, to determine the extent of collinearity between *X* variables (Table 2). *X* variables that were highly collinear with each other (correlation >0.500) are highlighted in grey (Table 2). *X* variables that were highly collinear included TGP and DMD, TGP and VFA/ACp/PRp, VFA and ACp/PRp/BTp, AC and PR/PRp, ACp and PRp/BTp, PR and PRp, PRp and BTp, BT and BTp, pH and BTp. These combinations were therefore avoided in multivariate meta-analysis equation development.

Correlation analysis was also used to examine potential correlations between *X* and *Y* variables (Table 2). The *X* variables moderately correlated (>0.300) with CH_4_d included DMD (−0.408), AC (0.405), ACp (0.350) and PR (−0.396), while *X* variables moderately correlated (>0.300) with CH_4_i included DMD (0.399), TGP (0.755), VFA (0.472), ACp (0.472), PRp (0.326) and BTp (0.315).

### 3.2. Univariate Meta-Analysis Models

Seventy-eight univariate equations to predict CH_4_d or CH_4_i were developed and evaluated with the variables presented in Table 2, in linear, quadratic or cubic form. Those with nonsignificant slope parameters or model fitting (fixed or random) problems were discarded, and the remaining equations (*n* = 22) were assessed on the evaluation dataset. On average, the CH_4_i outcome was predicted with higher CCC and lower RMSPE values compared to the CH_4_d outcome (Table 3). The best six performing equations have their model evaluation results presented in Table 3. The best performing univariate equations included the *X* variables ACp, PRp, DMD, VLp, VFA and TGP. The best performing univariate equations were those predicting CH_4_i with TGP as a driving *X* variable, with a CCC on the evaluation database of 0.644 (quadratic) and 0.650 (linear) (Table 3).

The results of univariate equation development (Table 3) agreed roughly with the correlation analysis (Table 2), where the variables most highly correlated with CH_4_i (TGP, VFA, ACp, VLp, DMD) and CH_4_d (DMD, AC, C2C3, PR) (Table 2) appeared in the best performing univariate equations (Table 3). Some differences were evident, for example in the *R*-values, which may be explained by the difference in approach (correlation across all data points vs. correlation within study). The best performing univariate equations (U6, U12) were as follows:

CH_4_d (mL CH_4_/g DM digested) = 58.52 (± 3.210) − 21.24 (± 3.045) × DMD (DM digestibility) (U6) (10)

CH_4_i (mL CH_4_/g DM incubated) = 3.00 (± 1.546) + 0.149 (± 0.005) × TGP (mL/g DM incubated) (U12) (11)

### 3.3. Multivariate Meta-Analysis Models

Seventy-two multivariate equations, to predict CH_4_d or CH_4_i, were developed and evaluated with the variables presented in Table 2, in linear combinations. Those with nonsignificant slope parameters, model fitting problems (fixed or random) or had multiple *X* variables which were previously deemed to be collinear (Table 2) were discarded, and the remaining equations (*n* = 29) were evaluated on the evaluation dataset. Evaluation of the top six performing multivariate equations (for each of CH_4_d and CH_4_i) is reported in Table 4. 

Best performing equations for CH_4_d included (1) equation M5 (CCC = 0.419) with DMD, VFA, PR, FT and VL as *X* variables, as well as (2) M6 (CCC = 0.425) with DMD and VFA as *X* variables (Table 4). Best performing equations for CH_4_i included (1) equation M11 (CCC = 0.438) with VFA and FT as *X* variables, and (2) equation M12 (CCC = 0.703) with PR, VL and TGP as *X* variables (Table 4). 

**Figure 1:**
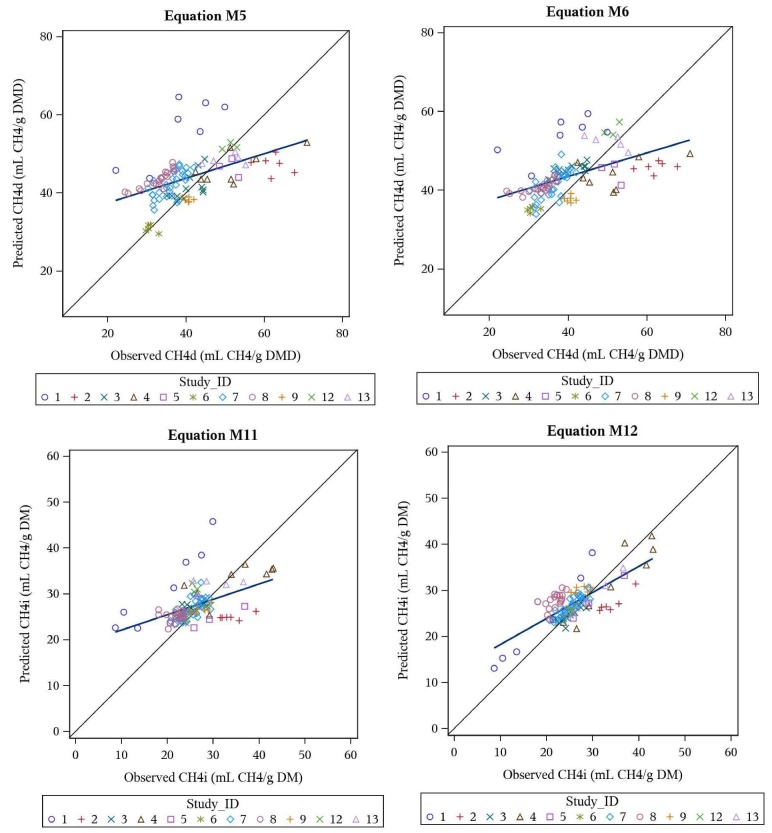
Predicted vs. observed plots for the top four performing meta-analysis equations M5 (CH_4_d), M6 (CH_4_d), M11 (CH_4_i) and M12 (CH_4_i), as evaluated on the evaluation dataset (*n* = 107).

The overall best performing equations (from univariate or multivariate origin, CH_4_d, CH_4_i) are presented in Table 5, and their predicted vs. observed plots are illustrated in Figure 1.

### 3.4. Support Vector Regression and Artificial Neural Network Models

**Figure 2:**
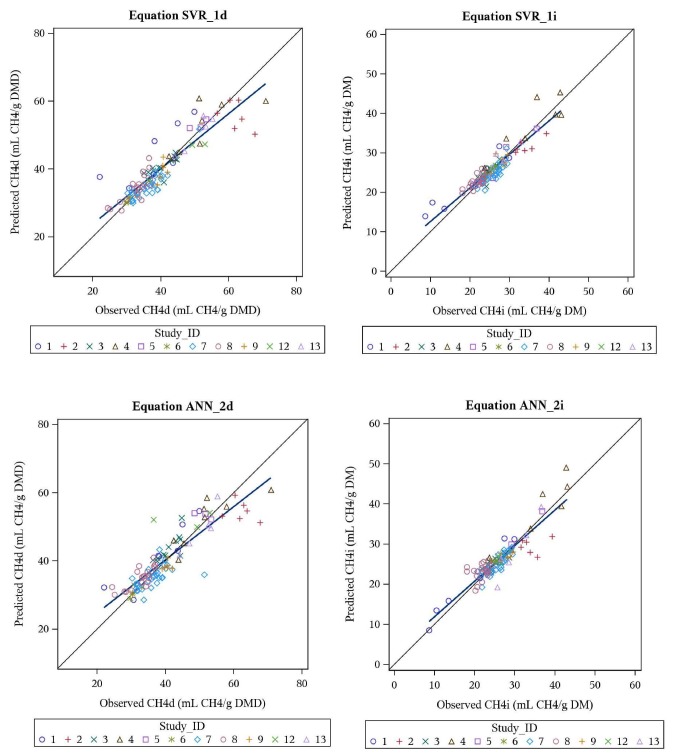
Predicted vs. observed plots for the machine learning equations SVR_1d (CH_4_d), SVR_1i (CH_4_i), ANN_2d (CH_4_d) and ANN_2i (CH_4_i), as evaluated on the evaluation dataset (*n* = 107).

Evaluation of SVR and ANN models developed are presented in Table 6. Both SVR and ANN models demonstrated high predictability on the test dataset, with CCC values >0.90 for both CH_4_d and CH_4_i. For comparison purposes, meta-analysis equations METd and METi were also developed, via meta-analysis, but included all *X* variables (in linear form, regardless of significance). The CCC values for these equations were 0.645 and 0.734, respectively (Table 6), indicating that the SVR and MLP models must consider a complex multiple-nonlinear response surface between the *X* variables and Y variables, in order to achieve substantially higher CCC values. The predicted vs. observed plots for these models are illustrated in Figure 2.

### 3.5. Behaviour Analysis—Machine Learning Models

Unlike the meta-analysis method that results in a predictive equation, the ML methods SVR and ANN do not have the same degree of transparency. To understand the causal pathways to obtain the predictive result, behaviour analysis was performed (Table 7) by systematically changing the inputs in isolation and determining the degree of change in the output prediction. This was performed at +10% and −10% to determine direction of change in the response variable. 

Results show (Table 7) that the models ANN_2i and SVR_1i (predicting CH_4_i) were highly sensitive to the *X* variables pH and TGP, to varying extents (dependent on the model and FT). Secondary to these variables, the CH_4_i predictions were sensitive to AC, PR, BT and DMD. Each model (ANN, SVR) demonstrated different sensitivity to these driving variables, and the sensitivity differed between the FT 1 (forage) and FT 2 (concentrate) substrates (Table 7).

For the models ANN_2d and SVR_1d (predicting CH_4_d), these were shown to be highly sensitive to the *X* variables pH, DMD, TGP, AC and BT (Table 7), again dependent on the method (ANN, SVR) and the FT (forage vs. concentrate). Driving variables that differed greatly in sensitivity between models (ANN vs. SVR) included pH (14% and 36% vs. −6% and 5% change with ±10%, FT = 1, CH_4_i), DMD (0% vs. −5% and 9% change with ±10%, FT = 1, CH_4_i) and AC (11% and −11% vs. 2% and 0% change with ±10%, FT = 1, CH_4_d) (Table 7), indicating that each approach fit the data slightly differently. 

Some responses had different directional effects in the different models. For example, increasing pH increased CH_4_i by 14% in ANN_2i, but decreased it by 6% in SVR_1i (FT = 1) (and similarly for FT = 2, pH increased CH_4_i in ANN_2i by 4% and by 30% in SVR_1i); increasing BT did not change CH_4_i in ANN_2i, but decreased CH_4_i by 6% in SVR1i (FT = 1); and increasing AC reduced CH_4_i by 3% in ANN_2i but increased CH_4_i by 7% in SVR_1i (FT = 2). Similar results were found for CH_4_d predictions, where, for example, increasing pH decreased CH_4_d with ANN_2d by 14%, but increased it by 9% with SVR_1d (FT = 1), and for FT = 2, raising pH increased CH_4_d by 11% with ANN_2d, and by 37% with SVR_1d. 

Some behaviour responses within the ML methods were also directionally different between FT. For example, CH_4_i (ANN_2i) increased as pH was increased (+14%, FT = 1), but also increased when pH was decreased (+36%), indicating a nonlinear/polynomial response surface. This is in contrast to when FT = 2, where increasing pH increased CH_4_i by 4%, and decreasing pH decreased CH_4_i by 24% (Table 7). For CH_4_i (SVR_1i, FT = 1), increasing pH decreased CH_4_i by 6%, while increasing pH increased CH_4_i by 5%. This is in contrast to when FT = 2, where increasing pH increased CH_4_i by 30%, and decreasing it decreased CH_4_i by 39%. Similar directional differences were observed for CH_4_d (FT = 1 vs. 2).

## 4. Discussion

To predict CH_4_ (in vivo or in vitro) based on stoichiometry principles alone and considering a H recovery of 100%, Hegarty and Nolan [8] proposed the equation: CH_4_ (mmol/L) = 0.5AC + 0.5BT − 0.25PR − 0.25VL (where all VFAs are expressed in mmol/L). Similarly, Moss et al. [9], considering a H recovery of 90%, proposed the equation: CH_4_ (mmol/L) = 0.45AC − 0.275PR + 0.40BT (where all VFAs are expressed in mmol/L). These equations reflect the net production of H as a result of AC and BT synthesis by rumen microbes and the net utilization of H as a result of PR and VL synthesis by rumen microbes during fermentation of feed. The resulting H is utilized by methanogens to reduce CO_2_ to H_2_O (CO_2_ + 8H → CH_4_ + 2H_2_O). However, predicting CH_4_ from the above stoichiometric equations is only valid if (1) these VFAs are the only end-products of fermentation, (2) no free H_2_ accumulates or escapes, (3) the microbial digestion process is strictly anaerobic, and (4) H_2_ is not used in other reactions (e.g., reduction of sulphates to sulphides, or saturation of double bonds in fatty acids) [8]. In practice, the production of CH_4_ will be less than the stoichiometry prediction given by the above equations, because these assumptions are generally not held. Jayanegara et al. [7] used both stoichiometric equations to predict CH_4_ from VFA concentrations in vitro, and found that indeed, the equations overpredicted CH_4_, likely due to a much lower observed H recovery (observed range of 28.9% to 56.2%) compared to the recoveries assumed by the models (100% and 90%). In agreement with Jayanegara et al. [7], when the stoichiometric equations [8,9] were applied to the current test dataset, CH_4_i was overpredicted (observed CH_4_i (mmol/L) = 11.6 ± 2.44, using [8], predicted CH_4_i = 16.2 ± 3.07; using [9], predicted CH_4_i = 14.0 ± 2.68) and had poor CCC evaluation statistics (0.135 and 0.227 for [8,9], respectively). For the test dataset, the average H_2_ recovery, calculated according to [31], was 80%, a value that is substantially lower than the theoretical recovery rates [8,9], and also different from those observed by Jayanegara et al. [7], indicating the potential value of an empirical approach, such as those developed in our work.

The objective of the current study was to utilize meta-analysis and ML methodologies to predict CH_4_ emissions from in vitro gas and VFA production data. Results of this work found that via meta-analysis, the best predictive equations of in vitro CH_4_d included the variables − DMD + VFA, or − DMD + VFA − PR − FT − VL (Equations M6 and M5 respectively, Table 5), while the best predictive equations of in vitro CH_4_i included the variables + VFA − FT, or − PR + VL + TGP (Equations M11 and M12, respectively, Table 5). The significant positive sign on VL in Equation M12 is concerning, as stoichiometrically, the production of VL utilizes H and therefore is associated with a lower CH_4_ emission. This illustrates a limitation of empirical modelling (whether it be meta-analysis or a ML), that the resulting equations strive to find the best statistical relationship to the data, regardless of biological principles. It is possible this could be related to the relatively small contribution to total VFA made by VL, or correlation with specific feed ingredient properties.

The best performing univariate equations (U6 (CH_4_d), U12 (CH_4_i)) were based on DMD and TGP, respectively. The correlation between CH_4_d and TGP was low (Table 2), indicating that the DMD correction to CH_4_i (CH_4_d) accounted for much of the strong relationship between CH_4_i and TGP. Such simple regressions may be used when VFA data are not reported, but would miss considerable variance explained by defining the type of VFA being produced (see multivariate equations). 

The models produced via ML methodologies, ANN and SVR, have much higher predictability (CCC, RMSPE analysis) of the CH_4_i and CH_4_d outcomes compared to the meta-analysis models. This was a result of the meta-analysis models being limited to including only significant *X* variables (*p* < 0.05), while the ML methodologies have no such limitation. As well, the ML methodologies mapped more complex response surfaces between multiple *X* variables and the *Y* variable, based on linear, radial or polynomial shapes. While this resulted in a greatly improved prediction on related (internal evaluation) data (Table 6), it may end up fitting noise or other unrelated data characteristics in the training dataset, resulting in a diminished predictability on unrelated data (external evaluation). Such an external evaluation would be a required next step to test the globalization ability of such ML models—in particular considering the relatively small size of the training dataset and the data hungry nature of ML models. 

Unsurprisingly, in both meta-analysis and ML models TGP was a significant driving variable, as an indicator of the overall extent of fermentation occurring in vitro. Directionally, the meta-analysis and ML methods agree, whereby increasing TGP increases CH_4_i and CH_4_d. The variable DMD was particularly relevant with the CH_4_d models, where increasing DMD resulted in a lower CH_4_d (Table 5 and Table 7). 

Interestingly, while pH did not appear in many highly significant meta-analysis equations (Table 3 and Table 4), it did appear to have a strong presence in the ML models, as illustrated by the behaviour analysis (Table 7). According to the pH dependent VFA stoichiometry of [32], an increase in ruminal pH causes a shift in soluble carbohydrate fermentation towards AC and away from PR and BT, and a shift in starch fermentation towards AC and BT and away from PR. For FT = 2 (concentrates), the ANN_2d and SVR_1d equations to predict CH_4_d show a tendency for CH_4_ to increase as pH increases (by 11% and 37%, respectively) (Table 7). In line, when pH is decreased, CH_4_d also decreased (Table 7). However, when FT = 1 (forages), and pH increases the ANN_2d prediction decreases (−14%), while the SVR_1d prediction increases (9%). For the ANN_2d equation, it is difficult to conceptualize where the −14% in CH_4_d comes from, aside from a nuance in the database. 

## 5. Conclusions

The current study successfully delivered models (using both meta-analysis and ML methodologies) which can be used to estimate CH_4_ production from in vitro fermentation systems. Meta-analysis results indicate that equations containing DMD, VFA, PR, FT and VL resulted in the best prediction of CH_4_ on an internal evaluation dataset of in vitro data. The ML models by far exceed the predictability achieved using meta-analysis methods, but should be evaluated on an external database to assess predictability and generalization potential on unrelated data, in particular given the limited database size and the data hungry nature of such ML methodologies. Between the ML methodologies assessed, ANN and SVR resulted in very similar predictive performance, but differences in fitting, as assessed by behaviour analysis, were evident. The models developed may be utilized to estimate CH_4_ emissions in vitro, in instances where total gas and VFA production, but not CH_4_, are measured.

## Figures and Tables

**Table 1:** Summary of the training (*n* = 247, 243) and internal evaluation (*n* = 107) datasets.

Variable ^1^	pH	DMD	TGP	CH_4_i	CH_4_d	VFA	AC	PR	BT	VL	C2C3
Training Dataset (Machine learning, *n* = 247)											
Mean	6.59	0.67	163	26.6	40.7	5.48	632.3	238.5	94.8	31.8	2.7
Median	6.64	0.68	160	25.3	38.0	5.33	632.2	237.7	94.4	31.8	2.7
Minimum	5.45	0.20	51	6.9	19.5	2.12	477.6	117.5	43.4	5.0	1.4
Maximum	6.78	0.91	276	50.8	71.5	9.79	812.1	346.9	181.3	79.3	7.0
Training Dataset (Meta-analysis, *n* = 243)											
Mean	6.59	0.67	164	26.7	40.6	5.50	631.7	239.2	94.9	31.9	2.7
Median	6.64	0.68	161	25.3	38.0	5.35	632.1	237.9	94.4	31.8	2.7
Minimum	5.45	0.22	71	11.2	19.5	2.12	477.6	117.5	60.0	10.3	1.4
Maximum	6.78	0.91	276	50.8	71.5	9.79	812.1	346.9	181.3	79.3	7.0
Evaluation Dataset (Machine learning/Meta-Analysis, *n* = 107)											
Mean	6.60	0.66	162	26.0	40.2	5.51	630.6	241.5	94.5	31.8	2.7
Median	6.64	0.68	162	25.4	37.9	5.25	629.0	238.5	96.4	32.5	2.7
Minimum	5.49	0.21	61	8.7	22.0	4.02	503.7	152.1	53.8	6.9	1.7
Maximum	6.84	0.87	246	43.0	70.9	9.81	787.5	333.1	178.9	60.8	5.1

^1^ Variables (units): pH = final pH in the incubation medium. DMD = apparent dry matter (DM) digestibility (g DM disappeared/g DM incubated). TGP = total gas production (mL gas/g DM incubated). CH_4_i = methane production (mL CH_4_/g DM incubated). CH_4_d = methane production (mL CH_4_/g DM apparently digested). VFA production (mmol total VFA/g DM incubated). AC = acetic acid (mmol AC/mol VFA). PR = propionic acid (mmol PR/mol VFA). BT = butyric acid (mmol BT/mol VFA). VL = valeric acid (mmol VL/mol VFA). C2C3 = acetate to propionate ratio.

**Table 2:** Correlation matrix (Pearson correlation coefficients, *R*) of dependent and independent variables, meta-analysis development dataset (*n* = 243) ^1,2^.

Variable	CH_4_d	CH_4_i	pH	DMD	TGP	VFA	AC	ACp	PR	PRp	BT	BTp	VL	VLp
**pH**	0.027	0.007												
**DMD**	−0.408	0.399	−0.060											
**TGP**	0.146	0.755	−0.206	0.707										
**VFA**	0.223	0.472	−0.437	0.282	0.648									
**AC**	0.405	−0.014	0.208	−0.474	−0.341	−0.159								
**ACp**	0.350	0.472	−0.340	0.136	0.544	0.944	0.168							
**PR**	−0.396	−0.045	−0.125	0.426	0.316	0.094	−0.863	−0.184						
**PRp**	−0.050	0.326	−0.431	0.439	0.663	0.817	−0.622	0.605	0.640					
**BT**	−0.035	0.045	−0.360	0.065	0.115	0.250	−0.279	0.136	−0.150	0.143				
**BTp**	0.103	0.315	−0.566	0.234	0.482	0.798	−0.294	0.679	−0.001	0.633	0.762			
**VL**	−0.113	0.223	0.273	0.369	0.096	0.007	−0.246	−0.071	0.023	0.016	0.145	0.071		
**VLp**	0.059	0.458	−0.021	0.439	0.435	0.555	−0.279	0.458	0.069	0.465	0.236	0.491	0.820	
**C2C3**	0.420	−0.038	0.070	−0.516	−0.373	−0.103	0.921	0.194	−0.924	−0.606	−0.078	−0.133	−0.186	−0.198

^1^ Statistical significance of *R* values (*n* = 243): *p* < 0.05 if |*R*| > 0.126, *p* < 0.01 if |*R*| > 0.165, *p* < 0.001 if |*R*| > 0.210. Light grey boxes (for pairwise correlations between *X* variables) show |*R*| > 0.500, light blue boxes (for correlations between *X* and *Y* variables) show |*R*| > 0.300, ^2^ Variables (units): CH_4_i = methane production (mL CH_4_/g DM incubated). CH_4_d = methane production (mL CH_4_/g DM apparently digested). pH = final pH in the incubation medium. DMD = apparent dry matter (DM) digestibility (g DM disappeared/g DM incubated). TGP = total gas production (mL gas/g DM incubated). VFA production (mmol total VFA/g DM incubated). AC = acetic acid (mmol AC/mol VFA). ACp = AC production (mmol AC/g DM incubated). PR = propionic acid (mmol PR/mol VFA). PRp = PR production (mmol PR/g DM incubated). BT = butyric acid (mmol BT/mol VFA). BTp = BT production (mmol BT/g DM incubated). VL = valeric acid (mmol VL/mol VFA). VLp = VL production (mmol VL/g DM incubated). C2C3 = acetate to propionate ratio.

**Table 3:** Univariate equations—meta-analysis model evaluation via root mean square prediction error (RMSPE) and concordance correlation coefficient (CCC) analysis on the internal evaluation (*n* = 107) database.

Equation ^1^	*Y*	*X*	Form	Mean ^2^	SEM	RMSPE, %	EB, %	ER, %	ED, %	CCC	*R*	*C_b_*
U1	CH_4_d	C2C3	Linear	44.7	0.37	25.7	19	2	79	0.113	0.194	0.579
U2	CH_4_d	PR	Quad	44.7	0.35	25.9	18	3	78	0.116	0.185	0.623
U3	CH_4_d	AC	Linear	44.8	0.29	25.6	20	1	79	0.124	0.228	0.541
U4	CH_4_d	PR	Linear	44.8	0.31	26.1	19	4	77	0.128	0.198	0.649
U5	CH_4_d	DMD	Quad	44.3	0.23	24.0	18	2	81	0.182	0.391	0.466
U6	CH_4_d	DMD	Linear	44.4	0.24	23.9	19	3	78	0.196	0.432	0.454
U7	CH_4_i	PRp	Quad	27.7	0.29	21.0	11	3	87	0.303	0.377	0.803
U8	CH_4_i	DMD	Cubic	28.2	0.19	21.8	15	4	80	0.305	0.375	0.813
U9	CH_4_i	VLp	Quad	28.0	0.26	20.7	14	0	85	0.314	0.420	0.747
U10	CH_4_i	VFA	Linear	27.4	0.16	20.9	7	7	86	0.346	0.390	0.889
U11	CH_4_i	TGP	Quad	27.2	0.33	15.5	10	1	90	0.644	0.717	0.898
U12	CH_4_i	TGP	Linear	27.3	0.31	15.5	10	0	89	0.650	0.717	0.906

^1^ Equation ID corresponds to equations presented in subsequent tables, with *Y* as the response (predicted) variable [either CH_4_i (observed mean 26.0 ± 0.53 mL CH_4_/g DM incubated) or CH_4_d (observed mean 40.2 ± 0.91 mL CH_4_/g DM apparently digested) and *X* as the explanatory variable (see Table 1 and Table 2 for abbreviations and units of each variable), ^2^ Mean = mean of predicted values; SEM = standard error of the mean of predicted values; RMSPE = root mean square prediction error expressed as a percentage of the observed mean; EB, ER and ED = error due to bias, regression and disturbance, respectively (all as % of total MSPE); CCC = concordance correlation coefficient; *R* = Pearson correlation coefficient (measure of precision); *C_b_* = bias correction factor (measure of accuracy).

**Table 4:** Linear multivariate equations—meta-analysis model evaluation via root mean square prediction error (RMSPE) and concordance correlation coefficient (CCC) analysis on the internal evaluation (*n* = 107) database.

Equation ^1^	*Y*	*X*	Mean ^2^	SEM	RMSPE, %	EB, %	ER, %	ED, %	CCC	*R*	*C_b_*
M1	CH_4_d	BTp, DMD	44.0	0.36	22.7	17	1	82	0.306	0.476	0.643
M2	CH_4_d	PRp, VLp, DMD	43.9	0.51	22.6	16	1	83	0.379	0.473	0.800
M3	CH_4_d	PR, VL, VFA, DMD	43.8	0.55	22.9	16	2	82	0.383	0.461	0.830
M4	CH_4_d	DMD, VFA, pH, PR	43.5	0.65	23.3	12	7	81	0.401	0.448	0.896
M5	CH_4_d	DMD, VFA, PR, FT, VL	43.8	0.58	22.5	16	2	82	0.419	0.492	0.853
M6	CH_4_d	DMD, VFA	43.5	0.53	21.7	14	1	85	0.425	0.516	0.823
M7	CH_4_i	pH, DMD, VLp, FT, BTp	28.5	0.34	21.1	21	3	76	0.407	0.496	0.833
M8	CH_4_i	pH, DMD, PRp, VLp, FT	28.4	0.33	20.8	21	2	78	0.410	0.520	0.826
M9	CH_4_i	pH, DMD, BTp, FT	28.3	0.32	20.2	20	1	79	0.428	0.520	0.823
M10	CH_4_i	DMD, VFA, FT	27.7	0.32	19.4	12	1	87	0.434	0.514	0.844
M11	CH_4_i	VFA, FT	27.4	0.37	19.8	8	5	87	0.438	0.484	0.905
M12	CH_4_i	PR, VL, TGP	27.2	0.40	14.8	11	0	89	0.703	0.752	0.936

^1^ Equation ID corresponds to equations presented in subsequent tables, with *Y* as the response (predicted) variable [either CH_4_i (observed mean 26.0 ± 0.53 mL CH_4_/g DM incubated) or CH_4_d (observed mean 40.2 ± 0.91 mL CH_4_/g DM apparently digested)] and *X* as the explanatory variables (see Table 1 and Table 2 for abbreviations and units of each variable; FT is feed type either forage or concentrate), ^2^ Mean = mean of predicted values; SEM = standard error of the mean of predicted values; RMSPE = root mean square prediction error expressed as a percentage of the observed mean; EB, ER and ED = error due to bias, regression and disturbance, respectively (all as % of total MSPE); CCC = concordance correlation coefficient; *R* = Pearson correlation coefficient (measure of precision); *C_b_* = bias correction factor (measure of accuracy).

**Table 5:** Best performing equations from the meta-analysis ^1^.

Equation ID	*Y*	Intercept	*X*1	*X*2	*X*3	*X*4	*X*5
M5	CH_4_d	76.35 (± 4.511)	−31.03 (± 3.922) × DMD	3.21 (± 0.352) × VFA	−0.094 (± 0.01202) × PR	−3.017 (± 1.460) (if FT = 1)	−0.133 (± 0.0380) × VL
M6	CH_4_d	51.35 (± 3.086)	−41.98 (± 3.429) × DMD	3.65 (± 0.390) × VFA			
M11	CH_4_i	15.8 (± 2.614)	3.06 (± 0.241) × VFA	−5.70 (± 1.028) (if FT = 1)			
M12	CH_4_i	11.58 (± 1.627)	−0.0633 (± 0.0057) × PR	0.0947 (± 0.01728) × VL	0.172 (± 0.0046) × TGP		

^1^ Variables (units): CH_4_i = methane production (mL CH_4_/g DM incubated). CH_4_d = methane production (mL CH_4_/g DM apparently digested). DMD = apparent dry matter (DM) digestibility (g DM disappeared/g DM incubated). TGP = total gas production (mL gas/g DM incubated). VFA production (mmol total VFA/g DM incubated). PR = propionic acid (mmol PR/mol VFA). VL = valeric acid (mmol VL/mol VFA). FT = feed type either forage or concentrate.

**Table 6:** Machine learning and meta-analysis (including all the *X* variables) model evaluation via root mean square prediction error (RMSPE) and concordance correlation coefficient (CCC) analysis on the internal evaluation (*n* = 107) database.

Equation ^1^	*Y*	*X*	Mean ^2^	SEM	RMSPE, %	EB, %	ER, %	ED, %	CCC	*R*	*C_b_*
SVR_1d	CH_4_d	all, nonlinear	40.2	0.82	9.9	0.5	0	99.5	0.899	0.905	0.994
SVR_1i	CH_4_i	all, nonlinear	26.1	0.49	8.3	0.6	0.1	99.3	0.917	0.920	0.997
ANN_2d	CH_4_d	all, nonlinear	40.5	0.80	9.5	0.5	0.8	98.7	0.907	0.915	0.991
ANN_2i	CH_4_i	all, nonlinear	26.0	0.52	9.1	0	2.9	97.1	0.906	0.906	1.000
METd	CH_4_d	all, linear	42.9	0.54	17.1	16	6	79	0.643	0.762	0.844
METi	CH_4_i	all, linear	27.2	0.40	14.0	12	0	88	0.734	0.782	0.939

^1^ Equation IDs with ‘d’ refer to CH_4_d, and ‘i’ refer to CH_4_i equations. METd and METi are meta-analysis equations. *Y* is the response (predicted) variable [either CH_4_i (observed mean 26.0 ± 0.53 mL CH_4_/g DM incubated) or CH_4_d (observed mean 40.2 ± 0.91 mL CH_4_/g DM apparently digested) and *X* are the explanatory variables (all variables included in this analysis for comparison purposes), ^2^ Mean = mean of predicted values; SEM = standard error of the mean of predicted values; RMSPE = root mean square prediction error expressed as a percentage of the observed mean; EB, ER and ED = error due to bias, regression and disturbance, respectively (all as % of total MSPE); CCC = concordance correlation coefficient; *R* = Pearson correlation coefficient (measure of precision); *C_b_* = bias correction factor (measure of accuracy).

**Table 7:** Behaviour analysis ^1^ of the artificial neural network (ANN) and support vector regression (SVR) models.

X-Variable	CH_4_i (on Average 25.5 and 36.3 mL CH_4_/g Dry Matter Incubated, for Forage and Concentrate, Respectively)				CH_4_d (on Average 40.0 and 46.4 mL CH_4_/g Dry Matter Apparently Digested for Forage and Concentrate, Respectively)			
	ANN (ANN_2i)		SVR (SVR_1i)		ANN (ANN_2d)		SVR (SVR_1d)	
Change in *X*-variable	+10% ^2^	−10% ^3^	+10% ^2^	−10% ^3^	+10% ^2^	−10% ^3^	+10% ^2^	−10% ^3^
**Feed type = forage (FT = 1)**								
pH	14%	36%	−6%	5%	−14%	7%	9%	35%
DMD	0%	0%	−5%	9%	−7%	18%	−13%	18%
TGP	12%	−10%	20%	−16%	15%	−8%	20%	−16%
Total VFA	−1%	1%	−1%	0%	−1%	1%	−1%	0%
Acetate (AC)	5%	11%	4%	3%	11%	−11%	2%	0%
Propionate (PR)	0%	0%	−2%	2%	−1%	1%	−1%	2%
Butyrate (BT)	0%	0%	−6%	6%	−1%	1%	−6%	8%
Valerate (VL)	−2%	2%	0%	−1%	−1%	1%	1%	−1%
C2C3	−1%	1%	1%	0%	−4%	4%	1%	0%
**Feed type = concentrate (FT = 2)**								
pH	4%	−24%	30%	−39%	11%	−23%	37%	−37%
DMD	−2%	−1%	5%	−2%	−8%	8%	−3%	5%
TGP	11%	−11%	12%	−10%	10%	−10%	9%	−8%
Total VFA	−4%	2%	−3%	2%	−2%	2%	−2%	1%
Acetate (AC)	−3%	3%	7%	−6%	3%	−3%	6%	−6%
Propionate (PR)	−3%	3%	−4%	4%	−3%	0%	−3%	3%
Butyrate (BT)	2%	−2%	−1%	1%	2%	−3%	−2%	2%
Valerate (VL)	1%	−1%	0%	0%	1%	−1%	0%	0%
C2C3	0%	−2%	1%	−1%	0%	0%	1%	−1%

^1^ Expected change (in %) in the predicted variable (either CH_4_i or CH_4_d), with a ±10% change in the driving variable. Changes in the predicted variable exceeding |10%| are highlighted in grey, ^2^ Increase of 10% in the explanatory variable, ^3^ Decrease of 10% in the explanatory variable.

## Appendix A

**Table A1:** Summary of variable abbreviations and units/descriptions.

Variable Abbreviation	Unit	Description
CH_4_i	mL CH_4_/g DM incubated	In vitro methane production
CH_4_d	mL CH_4_/g DM apparently digested	In vitro methane production
pH	-	Final pH in the incubation medium
DMD	g DM disappeared/g DM incubated	Apparent dry matter (DM) digestibility
TGP	mL gas/g DM incubated	Total gas production
VFA	mmol total VFA/g DM incubated	Total VFA production
AC	mmol AC/mol VFA	Acetic acid, proportion of total VFA
PR	mmol PR/mol VFA	Propionic acid, proportion of total VFA
BT	mmol BT/mol VFA	Butyric acid, proportion of total VFA
VL	mmol VL/mol VFA	Valeric acid, proportion of total VFA
ACp	mmol AC/g DM incubated	Acetic acid production
PRp	mmol PR/g DM incubated	Propionic acid production
BTp	mmol BT/g DM incubated	Butyric acid production
VLp	mmol VL/g DM incubated	Valeric acid production
C2C3	AC/PR	Acetate to propionate ratio

## References

[1] Pachauri R.K., Meyer L.A., IPCC, Core Writing Team (2014). Climate Change 2014 Synthesis Report. Contribution of Working Groups I, II and III to the Fifth Assessment Report of the Intergovernmental Panel on Climate Change.

[2] Calvo Buendia E., Tanabe K., Kranjc A., Baasansuren J., Fukuda M., Ngarize S., Osako A., Pyrozhenko Y., Shermanau P., Federici S., IPCC (2019). 2019 Refinement to the 2006 IPCC Guidelines for National Greenhouse Gas Inventories.

[3] Martin C., Morgavi D.P., Doreau M. (2010). Methane mitigation in ruminants: From microbe to the farm scale. Animal.

[4] Kumar S., Choudhury P.K., Carro M.D., Griffith G.W., Dagar S.S., Puniya M., Calabro S., Ravella S.R., Dhewa T., Upadhyay R.C. (2014). New aspects and strategies for methane mitigation from ruminants. Appl. Microbiol. Biotechnol..

[5] Hristov A.N., Oh J., Firkins J.L., Dijkstra J., Kebreab E., Waghorn G., Makkar H.P.S., Adesogan A.T., Yang W., Lee C. (2013). Special Topics—Mitigation of methane and nitrous oxide emissions from animal operations: I. A review of enteric methane mitigation options. J. Anim. Sci..

[6] Ellis L.L., Dijkstra J., Kebreab E., Bannink A., Odongo N.E., McBride B.W., France J. (2008). Aspects of rumen microbiology central to mechanistic modelling of methane production in cattle. J. Agric. Sci..

[7] Jayanegara A., Ikhsan I., Toharmat T. (2013). Assessment of methane estimation from volatile fatty acid stoichiometry in the rumen in vitro. J. Ind. Trop. Anim. Agric..

[8] Hegarty R.S., Nolan J.V., Makkar H.P.S., Vercoe P.E. (2007). Estimation of ruminal methane production from measurement of volatile fatty acid production. Measuring Methane Production from Ruminants.

[9] Moss A., Jouany J.-P., Newbold J. (2000). Methane production by ruminants: Its contribution to global warming. Ann. Zootech..

[10] McGeough E.J., O’Kiely P., O’Brien M., Kenny D.A. (2011). An evaluation of the methane output associated with high-moisture grains and silages using the in vitro total gas production technique. Anim. Prod. Sci..

[11] Navarro-Villa A., O’Brien M., López S., Boland T.M., O’Kiely P. (2011). Modifications of a gas production technique for assessing in vitro rumen methane production from feedstuffs. Anim. Feed Sci. Technol..

[12] Navarro-Villa A., O’Brien M., López S., Boland T.M., O’Kiely P. (2011). In vitro rumen methane output of red clover and perennial ryegrass assayed using the gas production technique (GPT). Anim. Feed Sci. Technol..

[13] Navarro-Villa A., O’Brien M., López S., Boland T.M., O’Kiely P. (2013). In vitro rumen methane output of grasses and grass silages differing in fermentation characteristics using the gas-production technique (GPT). Grass Forage Sci..

[14] Purcell P.J., O’Brien M., Boland T.M., O’Kiely P. (2011). In vitro rumen methane output of perennial ryegrass samples prepared by freeze drying or thermal drying (40 °C). Anim. Feed Sci. Technol..

[15] Purcell P.J., O’Brien M., Boland T.M., O’Donovan M., O’Kiely P. (2011). Impacts of herbage mass and sward allowance of perennial ryegrass sampled throughout the growing season on in vitro rumen methane production. Anim. Feed Sci. Technol..

[16] Purcell P.J., Boland T.M., O’Brien M., O’Kiely P. (2012). In vitro rumen methane output of forb species sampled in spring and summer. Agric. Food Sci..

[17] Purcell P.J., O’Brien M., Navarro-Villa A., Boland T.M., McEvoy M., Grogan D., O’Kiely P. (2012). In vitro rumen methane output of perennial ryegrass varieties and perennial grass species harvested throughout the growing season: In vitro rumen methane output of perennial grasses. Grass Forage Sci..

[18] Purcell P.J., Grant J., Boland T.M., Grogan D., O’Kiely P. (2012). The in vitro rumen methane output of perennial grass species and white clover varieties, and associative effects for their binary mixtures, evaluated using a batch-culture technique. Anim. Prod. Sci..

[19] Purcell P.J., Boland T.M., O’Kiely P. (2014). The effect of water-soluble carbohydrate concentration and type on in vitro rumen methane output of perennial ryegrass determined using a 24-hour batch-culture gas production technique. Irish J. Food Agric. Res..

[20] Navarro-Villa A., O’Brien M., López S., Boland T.M., O’Kiely P. Determination of the in vitro rumen methane output of contrasting feeds using the gas production technique (GPT).

[21] SAS Institute Inc. (2015). SAS/STAT^®^ 14.1 User’s Guide.

[22] St-Pierre N.R. (2001). Invited review: Integrating quantitative findings from multiple studies using mixed model methodology. J. Dairy Sci..

[23] Coelho L.P., Richert W., Brucher M. (2018). Building Machine Learning Systems with Python: Explore Machine Learning and Deep Learning Techniques for Building Intelligent Systems Using Scikit-Learn and TensorFlow.

[24] Buitinck L., Louppe G., Blondel M., Pedregosa F., Mueller A., Grisel O., Niculae V., Prettenhofer P., Gramfort A., Grobler J. API design for machine learning software: Experiences from the scikit-learn project. Proceedings of the European Conference on Machine Learning and Principles and Practice of Knowledge Discovery in Databases, Workshop on Languages for Data Mining and Machine Learning.

[25] Pedregosa F., Varoquaux G., Gramfort A., Michel V., Thirion B., Grisel O., Blondel M., Prettenhofer P., Weiss R., Dubourg V. (2011). Scikit-learn: Machine learning in Python. J. Mach. Learn. Res..

[26] Rossum G.V. (2018). Python Tutorial Release 3.6.4.

[27] Drucker H., Burges C.J.C., Kaufman L., Smola A.J., Vapnik V., Mozer M.C., Jordan M.I., Petsche T. (1997). Support vector regression machines. Advances in Neural Information Processing Systems 9.

[28] Paliwal M., Kumar U.A. (2009). Neural networks and statistical techniques: A review of applications. Expert Syst. Appl..

[29] Bibby J., Toutenburg T. (1977). Prediction and Improved Estimation in Linear Models.

[30] Lin L.I. (1989). A concordance correlation coefficient to evaluate reproducibility. Biometrics.

[31] Demeyer D., Van Nevel C. (1979). Protein fermentation and growth by rumen microbes. Ann. Rech. Vet..

[32] Bannink A., France J., López S., Gerrits W.J.J., Kebreab E., Tamminga S., Dijkstra J. (2008). Modelling the implications of feeding strategy on rumen fermentation and functioning of the rumen wall. Anim. Feed Sci. Technol..

